# Highly Specific Peptide-Mediated Cuvette-Form Localized Surface Plasmon Resonance (LSPR)-Based Fipronil Detection in Egg

**DOI:** 10.3390/bios12110914

**Published:** 2022-10-23

**Authors:** Jingon Yoo, Soobin Han, Bumjun Park, Sonam Sonwal, Munirah Alhammadi, Eunsu Kim, Sheik Aliya, Eun-Seon Lee, Tae-Joon Jeon, Mi-Hwa Oh, Yun Suk Huh

**Affiliations:** 1 Department of Biological Sciences and Bioengineering, Inha University, 100 Inha-ro, Michuhol-gu, Incheon 22212, Korea; 2 National Institute of Animal Science, Rural Development Administration, 1500, Kongjwipatjwi-ro, Iseo-myeon, Wanju-gun, Jeollabuk-do 55365, Korea

**Keywords:** biosensor, fipronil, localized surface plasmon resonance (LSPR), phage display, peptide screening

## Abstract

Herein, we have developed peptide-coated gold nanoparticles (AuNPs) based on localized surface plasmon resonance (LSPR) sensor chips that can detect fipronil with high sensitivity and selectivity. The phage display technique has been exploited for the screening of highly specific fipronil-binding peptides for the selective detection of the molecule. LSPR sensor chips are fabricated initially by attaching uniformly synthesized AuNPs on the glass substrate, followed by the addition of screened peptides. The parameters, such as the peptide concentration of 20 µg mL^−1^ and the reaction time of 30 min, are further optimized to maximize the efficacy of the fabricated LSPR sensor chips. The sensing analysis is performed systematically under standard fipronil solutions and spike samples from eggs. The developed sensor has shown excellent sensitivity towards both standard solutions and spike samples with limit of detection (LOD) values of 0.01 ppb, respectively. Significantly, the developed LSPR sensor chips offer distinct features, such as a facile fabrication approach, on-site sensing, rapid analysis, cost-effectiveness, and the possibility of mass production, in which the chips can be effectively used as a promising and potential on-site detection tool for the estimation of fipronil.

## 1. Introduction

Fipronil, an insecticide known as a type of *N*-phenylpyrazole, has been used in various agricultural and veterinary medicines since 1993 to control various vermin, such as crop pests, ants, cockroaches, and fleas from dogs and cats [1,2]. However, when fipronil is exposed to the human body through inhalation, intake, and skin contact, various symptoms, such as dizziness, sensory abnormalities, muscle weakness, difficulty breathing, coughing, asthma, skin irritation, erythema, itching, and fatigue, appear [3,4]. Due to its toxicity, the European Food Safety Administration banned the use of plant protection products containing fipronil, and the United States Environmental Protection Agency categorized fipronil as carcinogen-C, which is a class of human carcinogens [5,6]. Additionally, the maximum residue limit of fipronil for eggs in Korea is 0.02 mg/kg, which is around 20 ppb. Corresponding to the report from US EPA, the chronic reference dose (RfD) for fipronil is 0.0002 mg/kg/day based on the No Observable Adverse Effect Level (NOAEL) for chronic toxicity (0.5 ppm or 0.019 mg/kg/day) and an uncertainty factor of 100 [7]. Despite the restriction, a month after the distribution of eggs contaminated with fipronil in Europe in July 2017, fipronil was detected in eggs circulated in the Republic of Korea, causing great controversy [8]. Accordingly, the Ministry of Agriculture, Food, and Rural Affairs conducted a residual pesticide test on eggs from spawning farms, but the determination of the amount exposed at the site was difficult due to the lack of on-site fipronil analysis methods.

Fipronil in existing foods is assayed using highly sensitive analytical techniques such as gas chromatography–mass spectroscopy (GC–MS), liquid chromatography–tandem mass spectrometry (LC–MS/MS), and enzyme-linked immunosorbent assay (ELISA), which have advantages in that both quantitative and qualitative analysis is possible with very high sensitivity, but they also have several drawbacks as they require expensive equipment operated by experts and take a long time to extract, preprocess, and analyze results [9,10,11]. In addition, various methods for analyzing low concentrations of fipronil in food samples have not been developed. Hence, detection technology that can analyze fipronil easily and quickly is necessary to overcome these shortcomings and monitor products at the food distribution site. Localized surface plasmon resonance (LSPR) is one of the analysis methods that can overcome these shortcomings and analyze samples in the field. LSPR is a detection method using the characteristic that polarization occurs on the nanoparticle surface and vibrates locally when light with various wavelengths is irradiated on materials with local surfaces, such as gold- and silver-based metal nanoparticles [12,13]. Using this analysis method, the analysis is applied in various fields, such as biology, the environment, food, and medicine [14,15,16,17]. However, selectively detecting one specific substance with high sensitivity with LSPR sensors with only gold or silver nanoparticles attached is difficult [18]. Therefore, the sensitivity and selectivity of the LSPR sensor should be improved by screening the peptide that selectively binds to fipronil and attaches to the nanoparticles.

Methods of screening peptides that selectively respond to a specific substance represent another challenge, which is resolved by the phage display technique. In this technique, the phage displays the expressed peptides on its surface, followed by the development of a specific peptide that binds strongly to a particular target [19,20,21,22]. This technique has been widely used in the study of selecting functional peptides that bind to a target, because it has the advantage of amplifying phages with the desired peptide and analyzing the sequence to identify the amino acids of peptides [23,24]. For example, Cho, et al. discovered affinity peptides that bind specifically to interleukin-33 (IL-33) using the phage display technique and used the specific peptide to achieve drug development or allergy diagnosis [25]. Therefore, peptide screening that binds to specific substances, which is a huge advantage of the technique, can discover peptides that bind specifically to the residual pesticide, fipronil.

**Scheme 1:**
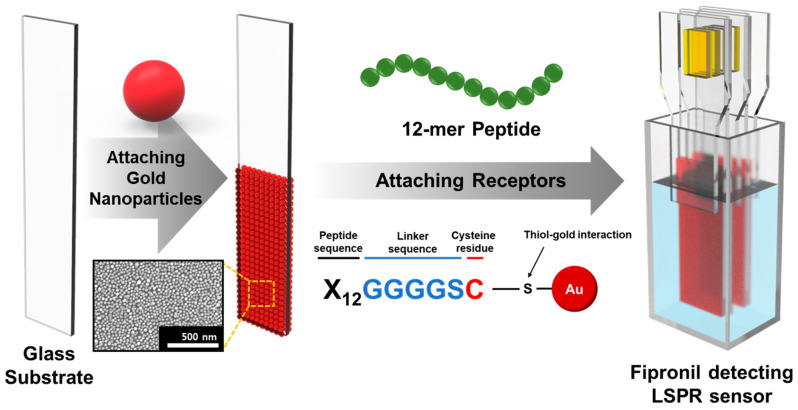
Schematic illustration of fipronil-specific LSPR sensor chip fabrication.

In this regard, in this study, an LSPR sensor with high sensitivity that can be used in the field is developed by screening peptides that selectively bind to fipronil and attaching them to the surfaces of gold nanoparticles, as shown in Scheme 1. Peptides that reacted selectively to fipronil were screened using a phage display technique using M13 phages with 12 mer peptides and were successfully attached to a uniformly synthesized, gold nanoparticle-coated LSPR chip. Subsequently, the reactivity of the fipronil LSPR sensor was confirmed to operate at high sensitivity after testing under various concentrations in standard and egg-based spike samples. Therefore, this selective fipronil-detectable LSPR sensor is expected to become a next-generation field diagnosis sensor in the food nutrition field to detect fipronil under various conditions effectively.

## 2. Materials and Methods

### 2.1. Materials

Gold (III) chloride trihydrate (HAuCl_4_. 3H_2_O, ≥99.9), fipronil (C_12_H_4_Cl_2_F_6_N_4_OS), (3-aminopropyl)triethoxysilane (APTES; C_9_H_23_NO_3_Si, ≥98.0%), sodium chloride (NaCl), tetracycline (C_22_H_24_N_2_O_8_), glycine (C_2_H_5_NO_2_), polyethylene glycol (PEG; C_2n_H_4n+2_O_n+1_), sodium iodide (NaI), and bovine serum albumin (BSA) were all purchased from the renowned company Sigma-Aldrich (St. Louis, MO, USA). Trisodium citrate dihydrate (C_6_H_9_Na_3_O_9_) was provided by Kanto Chemical Co., Inc. (Tokyo, Japan). Methyl alcohol (CH_3_OH, 99.5%) was purchased from Samchun Pure Chemical Co., Ltd. (Pyeongtaek-si, South Korea). LB broth was purchased from BD DIFCO. The maleic anhydride-activated plate (8-well strip) and phosphate-buffered saline (PBS; C_l2_H_3_K_2_Na_3_O_8_P_2_) were provided by Thermo Fisher (Waltham, MA, USA). The phage display library kit and *Escherichia coli* ER2738 strain were procured from New England Biolabs (Ipswich, MA, USA). The glass substrate was purchased from the Korea Testing & Research Institute (Gwacheon, Korea). The absorbance spectra were recorded on a V-770 UV–Visible/NIR Spectrophotometer (South Korea). The characterizations of the synthesized gold nanoparticles (AuNPs) were analyzed using field-emission scanning electron microscopy (FE–SEM; JITACHI S-4300 system) and field-emission transmission electron microscopy (FE-TEM; JEM2100F, JEOL Ltd., Peabody, MA, USA).

### 2.2. Screening of Fipronil-Binding Peptide through Phage Display Technique

First, 100 µL of 100 µM of fipronil in distilled water (DW) was treated on a maleic anhydride (MA)-activated 96-well plate overnight at room temperature. After immobilizing fipronil, 6 times, the plate was washed with 100 µL of 0.1% PBST. Next, 100 µL of phage library (1 × 1011 pfu mL^−1^ in PBS) was coated on the plate by incubating it for 1 h in an orbital shaker at a speed of 150 rpm to bind to the immobilized fipronil. Then, to remove unbound phages, the plate was rinsed 10 times with 200 µL of 0.1% PBST. Later, the plate was treated with 100 µL of 0.2 M glycine–HCl (pH 2.2) with 1 mg mL^−1^ BSA in an orbital shaker for 15 min at a speed of 150 rpm, for the elution of fipronil-bound phages. To the eluent solution, for the neutralization to take place, 15 µL of 1.0 M Tris–HCl (pH 9.1) was added. Then, it was transferred to a 1.5 mL microtube.

For the DNA sequencing, the overnight culture of *E. coli* with an optical density of 0.03~0.05 at 600 nm in LB medium was inoculated with a single plaque of *E. coli* (ER2738). The solution was incubated for 4.5 h at a speed of 200 rpm in a shaker incubator at 37 °C, followed by centrifugation for 1 min at 14,000 rpm at a temperature of 4 °C. Then, the supernatant was carefully transferred to a microtube, and then 500 μL was transferred to another tube along with 500 µL PEG–NaCl and was incubated for 15 min in an orbital shaker for DNA preparation. Another 200 µL of supernatant was used for ELISA testing. After 15 min of reaction, the solution was centrifuged at 14,000 rpm for 10 min at 4 °C. After completely removing the supernatant, dissolution of the pellet using 100 µL of iodide buffer was achieved; subsequently, 100% ethanol was added and incubation for 15 min was performed. Centrifugation for 10 min at 14,000 rpm (4 °C) was performed immediately after incubation. Then, the supernatant was discarded, and 70% ethanol was added, according to the same conditions. Again, the solution was centrifuged for 1 min at 14,000 rpm (4 °C). Finally, the pellets (phage DNA) were dried in a clean bench overnight, and then dissolved using TE buffer and stored at −20 °C. Later, DNA sequencing was performed at Solgent (Daejeon, Korea). As a result, four candidates of peptides were chosen for ELISA testing for sensitivity confirmation. Through ELISA testing, Peptide M38 showed the highest sensitivity for fipronil. However, when we tested all four peptides with an LSPR sensor, peptide M38 showed the highest sensitivity results. Therefore, it was chosen as the final fipronil receptor.

### 2.3. Fabrication of LSPR Sensor Chip

First, for the synthesis of AuNPs for plasmonic nanoparticles, AuNPs were synthesized through a Turkvich/Frens reaction. Here, 700 mL of DW was added to a reaction vessel, and 0.7 mL of 10% HAuCl_4_ solution was added to the water, heated up to 380 °C, and stirred at 200 rpm. When the solution started boiling, trisodium citrate solution was added and stirred till the solution changed to a red-wine color. Finally, the synthesized AuNPs were used in LSPR chip fabrication. To confirm the size and shape of the AuNPs, SEM and TEM analysis were performed.

The LSPR sensor chip’s glass substrate (8 mm × 50 mm) was immersed in a solution of methanol for 1 h. Then, it was subjected to sonication for 15–20 min, and then it was rinsed thrice thoroughly with deionized water. After the cleaning process, the glass substrate was immersed in a cuvette with 0.5% APTES solution and was kept in the oven at 60 °C for 1 h. Then, to remove the unbound and remaining APTES solution, the glass substrate was washed with deionized water five times. Then, the glass substrate was dipped in the AuNP solution overnight to allow AuNPs to attach to the glass chip as a single layer.

For selective fipronil detection, the peptide obtained from the phage display process was immobilized on the AuNPs of the plasmonic active substrate. After the dilution of the peptide with water, the LSPR substrate was dipped into the peptide solution for peptide immobilization and then washed with deionized water to remove unbound peptides. The optimization of the LSPR chip parameters was important to achieve high sensitivity and specificity. Therefore, two parameters were taken into consideration: first, the peptide concentration, and second, the peptide reaction time with the LSPR substrate. The final optimal conditions were chosen based on the peak shift observed by the UV–Vis spectrometer. Finally, to confirm the specificity of the peptide toward fipronil and reduce non-specific binding, the peptide-functionalized substrate was blocked using 1% BSA.

### 2.4. Performance of LSPR Sensor Chips on Standard Fipronil Solutions

The fipronil detection was performed on a standard solution with the fabricated LSPR sensor chip. After dipping the peptide-based LSPR sensor chip for approximately 10 min in the different concentrations of fipronil standard solution, the sensor chip was rinsed with double-distilled water to remove the fipronil that was not bonded with peptide receptors. The absorption spectra between 700 nm and 400 nm were recorded by the UV–Vis spectrophotometer. For more details of the optical setup of this study, the fabricated LSPR sensor chip was in a cuvette-based form, which fit excellently with the standard 1 mL volume cuvette. The light from the UV–Vis spectrophotometer was transmitted through the wide side of the LSPR chip (Figure S1). The standard samples were prepared in different dilutions, such as 0, 0.001, 0.01, 0.1, 1, 10, 100, 1000 ppb, to confirm the detection limit of this sensor.

### 2.5. Performance of LSPR Sensor Chips on Spiked Egg Samples

Fipronil-spiked egg samples were prepared to check whether fipronil could be detected in actual food. Eggs were purchased at the local mart and were ground with a mixer; then, 1 mL homogenized egg samples were spiked with various fipronil concentrations. Subsequently, LSPR sensor chips were reacted with fipronil-spiked egg samples using the detection process described above.

## 3. Results and Discussion

### 3.1. Screening of Fipronil-Specific Binding Peptide through Phage Display Technique

**Figure 1:**
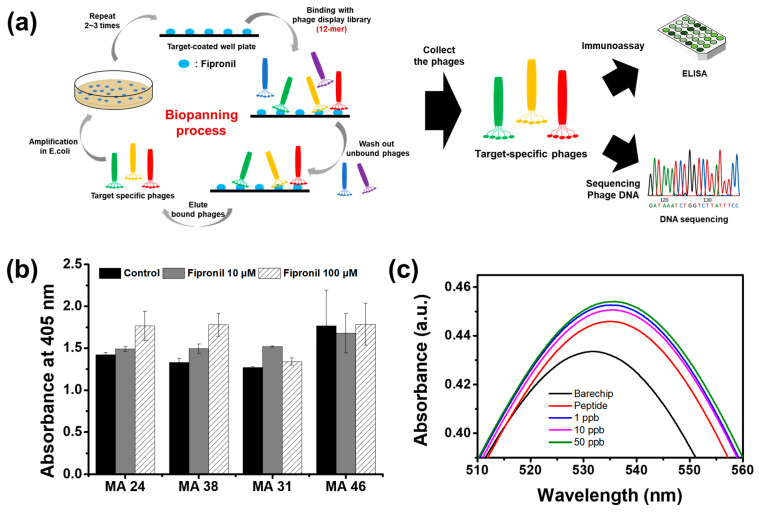
Screening of specific binding peptides of fipronil through phage display technique: (**a**) biopanning process and identification of fipronil-specific peptides, (**b**) screening of phage candidate group displaying peptides specific for fipronil, and (**c**) detection of fipronil using optimized LSPR sensor chip.

The phage display technique was employed to screen for the peptide receptor that selectively bound to fipronil (Figure 1a). This technique can be divided into three major steps: biopanning, DNA sequencing, and enzyme-linked immunoassay (ELISA) analysis. Initially, biopanning is the process used to screen the M13 phages that selectively bind to fipronil, fixed on a well plate by injecting a phage library containing various types of phages [23]. Phages that selectively bind to fipronil are then separated through an elution process, followed by amplification, preparing for the next round of biopanning. In the case of screening phages for selective binding to fipronil, three rounds of biopanning are performed. Subsequently, DNA sequencing is performed using the eluted phages from the third round, followed by ELISA analysis using sequences with many overlaps [26].

ELISA analysis progressed using four phage candidate groups selected from DNA sequencing. As shown in Figure 1b, the phage candidate groups of MA24 and MA38 showed higher absolute and correlated values as the fipronil concentration was increased. The sequence results of the two phage candidate groups were different, as shown in Table S1. Among the MA24 and MA38 sequences, the degree to which a peak shift occurred in screened peptides that reacted more specifically to fipronil was confirmed (Figure 1c). Therefore, the peptide that could be applied to the LSPR sensor chips was screened for MA38.

### 3.2. Fabrication of Fipronil-Specific Localized Surface Plasmon Resonance Sensor

The LSPR technique detects small molecules in samples with a low concentration very efficiently and has attracted attention for application in sensing pesticides. The LSPR frequency is affected by factors such as the size, shape, and dielectric and interparticle spacing characteristics of the used material, along with the dielectric properties of the environmental surroundings of the nanoparticles [27]. Usually, in LSPR sensors, silver (Ag) or AuNPs are used. As Ag particles are known for their lower stability and complex chemical synthesis, herein, AuNPs were used in this study as they are chemically highly stable.

Au NPs were synthesized through a Turkevich–Frens reaction system in which HAuCl4 was reduced using sodium citrate [28,29]. The shape and size of AuNPs are dependent on the ratio of gold-reductant concentrations. This synthesis technique is simple, cost-effective, and highly reproducible [30]. In particular, the synthesis of uniform nanoparticles of controlled size is an important factor to improve the performance of AuNP-based biosensors [31]. In the case of LSPR sensor chips, previous research proved that sensitive LSPR signals could be obtained as the generation of hotspots that can increase the surface charge when the size of AuNPs was decreased [32]. Based on the theory, small, uniformly sized AuNPs were synthesized and fabricated on the LSPR chip. They were analyzed through FE-TEM (Figure S2). The AuNPs used in the LSPR sensor chip had a generally uniform size of 12.32 ± 1.01 nm (Figure S2a) with a lattice spacing of 0.23 nm, which was matched with the (111) crystal plane, indicating that the synthesized AuNPs were suitable (Figure S2b) [33]. The LSPR chip’s size measurements were precisely maintained according to the size of the UV cuvette cell to fabricate a highly stable and robust cuvette-type LSPR sensor for easy handling while testing with a UV–Vis spectrophotometer.

**Figure 2:**
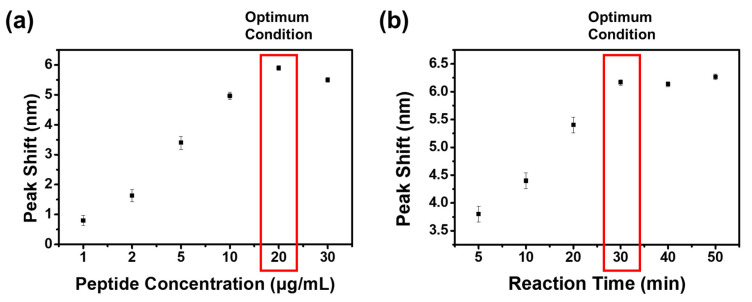
Optimization of LSPR sensor chip: (**a**) effect of peptide concentration on detection of fipronil, and (**b**) optimal reaction time with 20 µg mL^−1^ of peptide.

LSPR sensor chips that selectively detect fipronil were fabricated initially by coating pre-synthesized 12-nm-sized AuNPs on the glass substrate, followed by the attachment of screened peptide receptors that selectively combine with fipronil. The optimal conditions of the LSPR sensor chips were confirmed by adjusting the concentration of the screened fipronil-binding peptides and the reaction time (Figure 2). Optimization of the peptide concentration was required not only to set the sensitivity and selectivity of the sensor chips but also to prevent false positives by suppressing the non-specific binding, as the peptides could completely cover the surfaces of the AuNPs. Therefore, as shown in Figure 2a, the effect of the peptide concentration was verified by measuring the LSPR peak shifts after the reaction between LSPR sensor chips and peptides under various concentrations (1.0 µg mL^−1^ to 30.0 µg mL^−1^). The results indicated that a peptide concentration of up to 20.0 µg mL^−1^ showed peak shift changes up to 5.9 nm, whereas the peak shift was rather decreased at a higher concentration over 20.0 µg mL^−1^, which was not an appropriate condition for the LSPR sensor chips. Optimization of the reaction time to attach the maximum amount of peptides on the surfaces of the LSPR sensor chips was further analyzed (Figure 2b). The results showed that the LSPR peaks significantly increased as the time increased up to 30 min, while a saturation curve without a large difference in peaks was formed after 30 min. As a result, the optimum conditions of LSPR sensor chips to selectively detect fipronil were determined as a peptide concentration of 20.0 µg mL^−1^ and reaction time of 30 min.

### 3.3. Detection of Fipronil Based on Standard Solutions and Spike Samples

**Figure 3:**
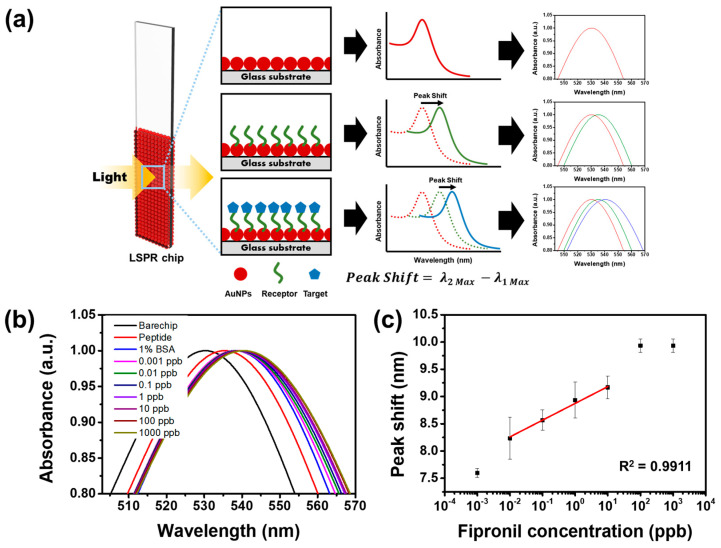
Detection of fipronil standard solution with high sensitivity through optimized LSPR sensor chip: (**a**) peak shift observed as the Au NPs bind, peptide binds, and fipronil binds; (**b**) sensitivity of LSPR sensor chips under various concentrations of fipronil standard solutions, confirmed with UV–Vis spectrum; and (**c**) changes in the spectrum peak at different concentrations of fipronil standard solution reacted on the chip.

Fipronil-specific LSPR sensor chips were operated by calculating the amount of peak shift that occurred, as shown in Figure 3a. When the glass substrate of the chip was coated with bare AuNPs, a peak appeared at the wavelength of 535 nm. Comparably, a wide red-shift of 50 nm, which was 540 nm of the characteristic peak, occurred when fipronil-specific binding peptides were attached to AuNPs due to the collective oscillation of conduction electrons [34]. From the 540 nm peak, the red-shift occurred once more, as fipronil was attached with the specific binding peptide on the surfaces of the AuNPs, in which a stronger peak difference occurred as the concentration of fipronil became higher.

The performance of the fabricated LSPR sensor chips that could selectively detect fipronil was tested on standard fipronil solutions and spiked samples from eggs. First, chip analysis at a broad range from 1 ppt to 1 ppm was performed to confirm the limit of detection (LOD) value. As shown in Figure 3b, the plasmon spectrum started showing a red shift as the concentration of fipronil was increased, which revealed that the fabricated LSPR sensor chips were highly sensitive. For quantitative analysis, a linear curve using the peak shift value was plotted as a function of the concentration change from the standard fipronil concentration range, and a dynamic range was achieved (Figure 3c). Considering the achieved dynamic range, the fipronil-specific LSPR sensor chip showed an LOD value of 0.01 ppb. The results indicated that the fabricated LSPR sensor chips performed excellently, with high sensitivity and stability for the detection of fipronil under standard solutions.

**Figure 4:**
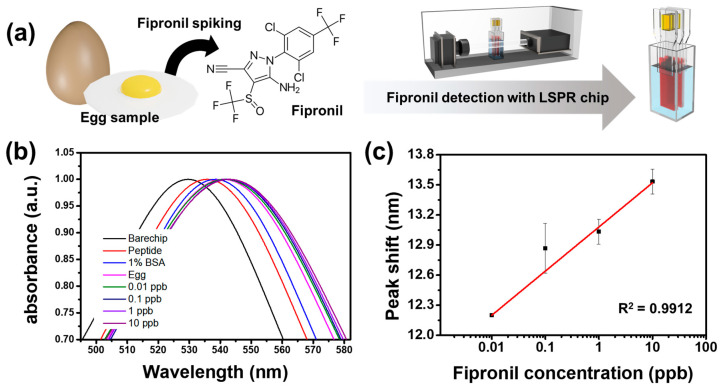
Detection of fipronil-included spiked egg samples using optimized LSPR sensor chips: (**a**) schematic illustration of the experiment, (**b**) sensitivity of LSPR sensor chips under various concentrations of spike samples, and (**c**) changes in the spectrum peak at different concentrations of spike samples reacted on the chip.

Subsequently, the performance of the fabricated fipronil-specific LSPR sensor chips on spiked egg samples was further tested to evaluate the possibility of on-site detection and real-time application (Figure 4). The spiked egg samples were prepared with homogenized whole egg, including white and yolk, in this study. The homogenization step was performed by vigorously shaking the sample and blending it with a vortex mixer. Then, 700 uL of the homogenized fipronil spiked egg sample was placed in a universal standard 1 mL volume cuvette with the fabricated LSPR sensor chips by adding different standard concentrations of fipronil (1 ppt to 1 ppm) to the individual egg, as shown in Figure 4a. The results showed similar behavior to standard solutions in that the red peak shift was observed due to the change in the plasmon resonance of the dielectric material in the presence of different fipronil concentrations (Figure 4b). Moreover, the linear dynamic range was found to be identical to that of the standard solution, with the LOD value of 0.01 ppb, as shown in Figure 4c. There was no change in the absorbance values, indicating that there was no matrix effect, even when using the whole egg without dilution. Therefore, the presented sensor is promising and could be used for a wide range of on-site detection applications in various food industries.

## 4. Conclusions

In summary, fipronil-specific cuvette-type LSPR sensor chips were economically fabricated with high specificity for the rapid and on-site detection of fipronil. Peptides that selectively bind to fipronil were initially screened through the phage display technique, followed by verification through ELISA analysis. Screened peptides were then attached to the surfaces of AuNP-based LSPR sensor chips to improve the sensitivity and selectivity towards fipronil. Both under standard solutions and spiked samples from eggs, LSPR sensor chips showed a red shift with the LOD value of 0.01 ppb, respectively. The detection limit is far more sensitive than that of the other reported sensors. Thus, the fabricated novel biosensor chips permitted the accurate and convenient measurement of fipronil owing to their outstanding advantages, such as simple and low-cost fabrication, small sample utilization, and output in a short period, with the high-performance, ultra-sensitive detection of fipronil in eggs and various types of food samples. Further, we anticipate that the developed LSPR sensor chips will open up pioneering approaches, novel insights, and major developments for the fabrication of other on-site detection sensors to detect various other harmful pesticides and food additives.

## Data Availability

Not applicable.

## Supplementary Materials

The following supporting information can be downloaded at: https://www.mdpi.com/article/10.3390/bios12110914/s1, Figure S1: Schematic illustration of the optical setup of this study. The light from the UV-Vis spectrophotometer transmitted through the wide side of the LSPR chip, due to the plasmonic resonance effects, peak shift occurs by the difference of molecule binding on the surface of the LSPR sensor chip; Table S1: Peptides derived by phage display technique. Peptide sequences of (a) MA24, (b) MA38, (c) MA31, and (d) MA46 deduced from DNA sequencing; Figure S2: Gold nanoparticle synthesis and characterization: (a) A TEM image showing spherical morphology and physical diameter of AuNPs, (b) size distribution graph of fabricated AuNPs, and (c) a magnified view clearly revealing the lattice structure of AuNPs.
