# Supplementary material for: Highly Specific Peptide-Mediated Cuvette-Form Localized Surface Plasmon Resonance (LSPR)-Based Fipronil Detection in Egg
Source: Biosensors (Basel). 2022 Oct 23;12(11):914. doi: 10.3390/bios12110914 (PMC9687660; doi:10.3390/bios12110914)
Supplement: Supplementary file 1 [file biosensors-12-00914-s001.zip › biosensors-1941586-supplementary.pdf]

Highly Specific Peptide-Mediated Cuvette-Form Localized Surface Plasmon Resonance (LSPR)-Based Fipronil Detection in Egg

Jingon Yoo [1,†], Soobin Han [1,†], Bumjun Park [1], Sonam Sonwal [1], Munirah Alhammadi [1], Eunsu Kim [1], Sheik Aliya [1], Eun-Seon Lee [2], Tae-Joon Jeon [1,*], Mi-Hwa Oh [2,*] and Yun Suk Huh [1,*]

[1] Department of Biological Sciences and Bioengineering, Inha University, 100 Inha-ro, Michuhol-gu, Incheon 22212, Korea
[2] National Institute of Animal Science, Rural Development Administration, 1500, Kongjwipatjwi-ro, Iseo-myeon, Wanju-gun, Jeollabuk-do 55365, Korea
* Correspondence: tjjeon@inha.ac.kr (T.-J.J.); moh@korea.kr (M.-H.O.); yunsuk.huh@inha.ac.kr (Y.S.H.)
† These authors contributed equally to this work.

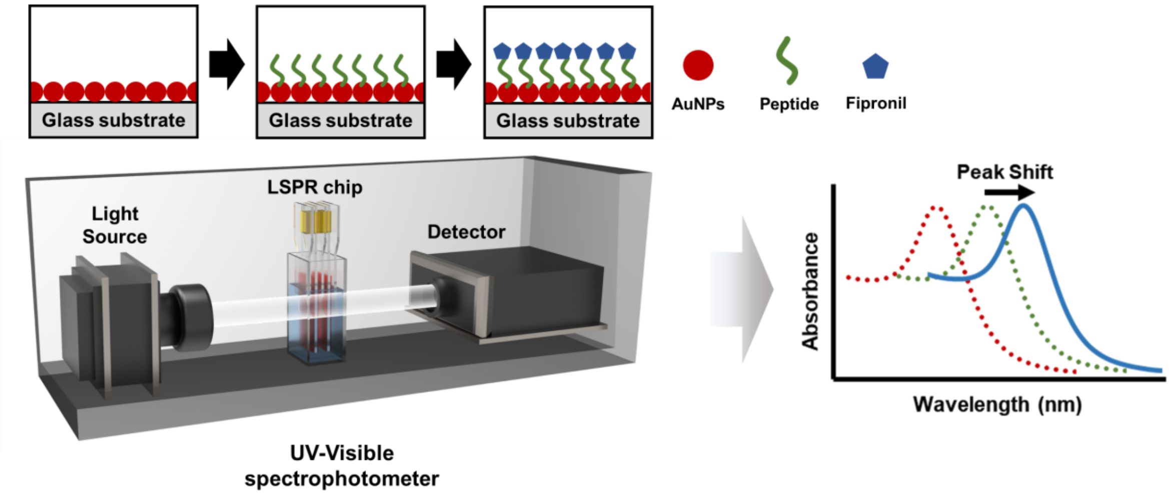

Figure S1. Schematic illustration of the optical setup of this study. The light from the UV-Vis spectrophotometer transmitted through the wide side of the LSPR chip, due to the plasmonic resonance effects, peak shift occurs by the difference of molecule binding on the surface of the LSPR sensor chip.

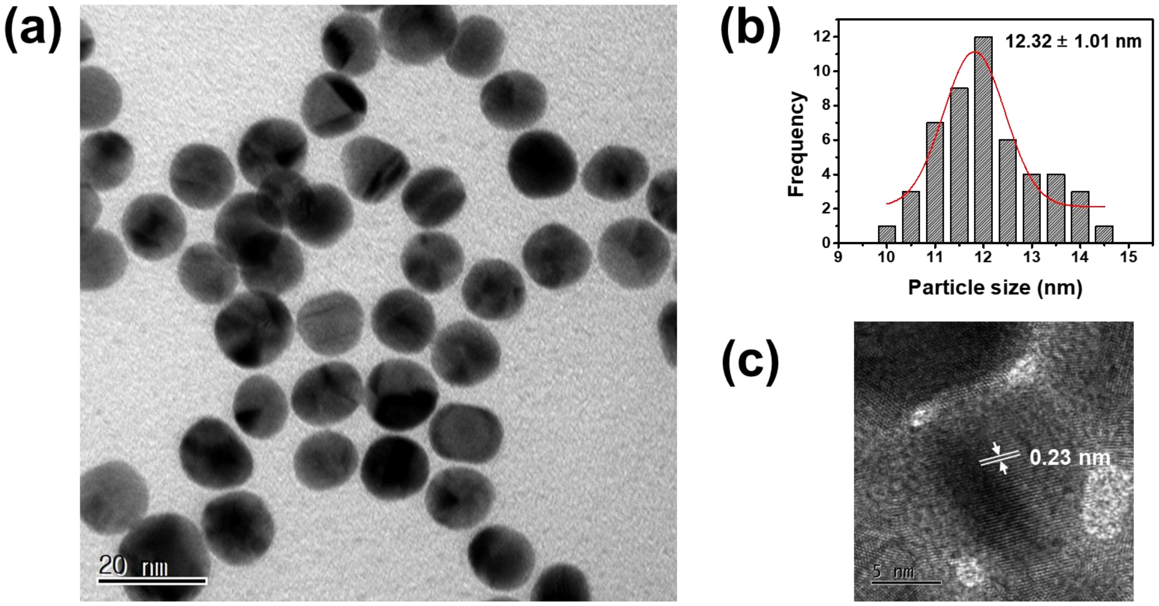

Figure S2. Gold nanoparticle synthesis and characterization: (a) A TEM image showing spherical morphology and physical diameter of AuNPs, (b) size distribution graph of fabricated AuNPs, and (c) a magnified view clearly revealing the lattice structure of AuNPs.

Table S1. Peptides derived by phage display technique. Peptide sequences of (a) MA24, (b) MA38, (c) MA31, and (d) MA46 deduced from DNA sequencing.

Phage clone	Peptide sequence	Frequency
(a) MA24	TPPSSNSYDWLV	4/48
(b) MA38	SNTALPSKFYGY	3/48
(c) MA31	INQDARTMVMVP	1/48
(d) MA46	GHQGHWYGMFRA	1/48
